# High Contributions of Secondary Inorganic Aerosols to PM_2.5_ under Polluted Levels at a Regional Station in Northern China

**DOI:** 10.3390/ijerph13121202

**Published:** 2016-12-15

**Authors:** Yang Li, Jun Tao, Leiming Zhang, Xiaofang Jia, Yunfei Wu

**Affiliations:** 1 Meteorological Observation Center, China Meteorological Administration, Beijing 100081, China; jiaxiaofangstar@126.com; 2 South China Institute of Environmental Sciences, Ministry of Environmental Protection, Guangzhou 510655, China; taojun@scies.org; 3 Air Quality Research Division, Science and Technology Branch, Environment and Climate Change Canada, Toronto, ON M3H 5T4, Canada; leiming.zhang@canada.ca; 4 Key Laboratory of Regional Climate-Environment for Temperate East Asia (RCE-TEA), Institute of Atmospheric Physics, Chinese Academy of Sciences, Beijing 100029, China; wuyf@mail.iap.ac.cn

**Keywords:** chemical composition, backward trajectory analysis, regional transport, potential source contribution function

## Abstract

Daily PM_2.5_ samples were collected at Shangdianzi (SDZ) regional site in Beijing–Tianjin–Hebei (BTH) region in 2015. Samples were subject to chemical analysis for organic carbon (OC), elemental carbon (EC), and major water-soluble inorganic ions. The annual average PM_2.5_ mass concentration was 53 ± 36 μg·m^−3^ with the highest seasonal average concentration in spring and the lowest in summer. Water-soluble inorganic ions and carbonaceous aerosols accounted for 34% ± 15% and 33% ± 9%, respectively, of PM_2.5_ mass on annual average. The excellent, good, lightly polluted, moderately polluted, and heavily polluted days based on the Air Quality Index (AQI) of PM_2.5_ accounted for 40%, 42%, 11%, 4%, and 3%, respectively, of the year. The sum of the average concentration of sulfate, nitrate, and ammonium (SNA) increased from 4.2 ± 2.9 μg·m^−3^ during excellent days to 85.9 ± 22.4 μg·m^−3^ during heavily polluted days, and their contributions to PM_2.5_ increased from 15% ± 8% to 49% ± 10% accordingly. In contrast, the average concentration of carbonaceous aerosols increased from 9.2 ± 2.8 μg·m^−3^ to 51.2 ± 14.1 μg·m^−3^, and their contributions to PM_2.5_ decreased from 34% ± 6% to 29% ± 7%. Potential source contribution function (PSCF) analysis revealed that the major sources for high PM_2.5_ and its dominant chemical components were within the area mainly covering Shandong, Henan, and Hebei provinces. Regional pollutant transport from Shanxi province and Inner Mongolia autonomous region located in the west direction of SDZ was also important during the heating season.

## 1. Introduction

High levels of fine particles (PM_2.5_, with an aerodynamic diameter ≤2.5 μm) have been a common phenomenon in recent decades in China [1]. The annual average PM_2.5_ mass concentration of 74 major Chinese cities reached up to 72 μg·m^−3^ in 2013, which was two times of the National Ambient Air Quality Standards (NAAQS) for annual PM_2.5_ (35 μg·m^−3^). The annual average PM_2.5_ exceeded the NAAQS in about 96% of the 74 cities. Among these cities, 13 are in the Beijing–Tianjin–Hebei (BTH) region, which had an annual average PM_2.5_ of 106 μg·m^−3^, much higher than that in Yangtze River Delta (YRD) region (25 cities with an average of 67 μg·m^−3^) and Pearl River Delta (PRD) region (9 cities with an average of 47 μg·m^−3^). The BTH region was evidently the most polluted region in China. PM_2.5_ has important effects on human health [2,3]. Thus, a thorough knowledge of the characteristics of PM_2.5_ chemical composition and sources in BTH is needed for assessing the effectiveness of the existing pollution control measures and for making future control measures to alleviate PM_2.5_ pollution.

Many studies have been conducted in BTH, especially in Beijing, in the past decade to investigate chemical compositions and sources of PM_2.5_ [4,5,6,7,8,9,10,11]. Most studies were short-term (e.g., less than one year) with only one study covering every day of one-year in urban Beijing [5]. Several studies revealed varying characteristics of PM_2.5_ chemical compositions under different pollution levels in urban Beijing [12,13], a knowledge that is needed for preventing extreme PM_2.5_ pollution events.

To date, synchronic and continuous measurements of chemically-resolved PM_2.5_ data in BTH are still rare, except at a rural regional representative site—Shangdianzi (SDZ) located far northeast of the megacities (Beijing, Tianjin, and Shijiazhuang) [14]. Despite being a rural site, the highest seasonal averages of the dominant chemical components (e.g., SO_4_^2−^, NO_3_^−^, NH_4_^+^, OC (organic carbon), and EC (elemental carbon)) in PM_2.5_ at SDZ station were at similar levels to those at the urban sites in major cities such as Beijing, Tianjin, and Chengde [14]. Thus, analyzing chemically-resolved PM_2.5_ data at SDZ can to some extent provide regional characteristics of PM_2.5_ pollution in BTH. For this purpose, one-year continuous measurements of chemically-resolved PM_2.5_ data at SDZ were thoroughly analyzed to (1) systemically characterize PM_2.5_ levels and the dominant chemical components; (2) compare the chemical composition changes under different PM_2.5_ pollution levels; and (3) identify the possible source regions for dominant chemical components.

## 2. Materials and Methods

### 2.1. Site Description

**Figure 1:**
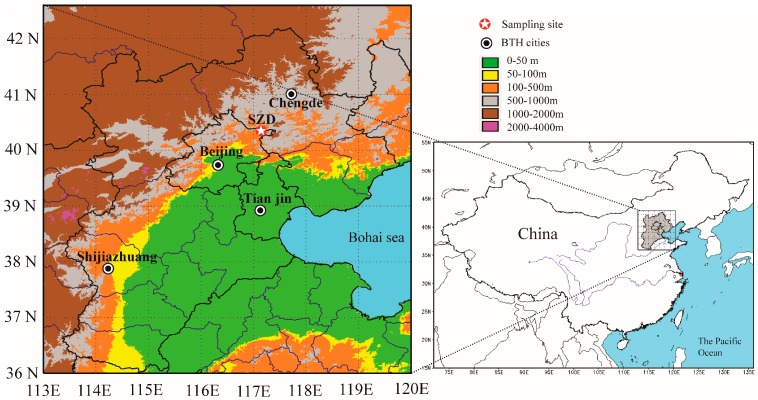
The sampling location at Shangdianzi (SDZ) regional station in Beijing–Tianjin–Hebei (BTH).

The international Global Atmosphere Watch programme (GAW) was launched in 1989 to monitor atmosphere chemical compositions. At present, the GAW network includes more than 400 regional stations and 31 global stations which are mostly located in remote areas. SDZ station was the only regional station of GAW in BTH. SDZ station (40°39′ N, 117°07′ E, 293 m a.s.l.) is located in the north boundary of the North China Plain and is 100 km northeast of urban Beijing (Figure 1). The instruments used in this study were installed on the roof (7 m above ground) of a building at SDZ station. More information about this station can be found in previous studies [14,15,16]. Major meteorological parameters including temperature and relative humidity (probe model HMP155), precipitation (rain gauge model SL3-1), and sunshine duration (sunshine recorder model XE66FJ-1) were observed at this site.

### 2.2. Sampling

PM_2.5_ samples were collected using a particulate sampler (BGI Incorporated, Waltham, MA, USA, Model PQ200) at a flow rate of 16.7 L·min^−1^. Samples were collected on 47 mm quartz fiber filter (Whatman QM-A). Sampling duration was 23 h starting at 9:00 local time each day and ending at 8:00 the following day. A total of 348 PM_2.5_ samples and 24 blank samples were collected from January to December 2015. The sampling for the whole year was divided into four seasons: spring (March–May), summer (June–August), autumn (September–November), and winter (January, February, and December). Filter backgrounds were used to account for any artifacts caused by gas absorption, evaporation of semi-volatile compounds, and background filters. The net mass of samples ranged from 335 to 5552 μg, while the net mass of the blank filters accounted for about ±20 μg. The uncertainty of PM_2.5_ mass concentration ranged from ±0.4% to ±6%. Thus, the uncertainties caused by the Quartz filters are negligible. The aerosol-loaded filter samples were stored in a freezer at −18 °C before analysis to prevent the volatilization of particles. Quartz filters were also measured gravimetrically for calculating PM_2.5_ mass concentration.

### 2.3. Chemical Analysis

OC and EC fractions of each quartz filter were analyzed using a DRI model 2001 carbon analyzer (Atmoslytic, Inc., Calabasas, CA, USA) following the IMPROVE_A thermal/optical reflectance (TOR) protocol [17]. Eight water-soluble inorganic ions (SO_4_^2−^, NO_3_^−^, Cl^−^, Na^+^, NH_4_^+^, K^+^, Mg^2+^, and Ca^2+^) of each quartz filter were determined by ion chromatography (Dionex ICS-600, Dionex Corp, Sunnyvale, CA, USA) [18]. Procedural blanks and average field blank values were subtracted from sample concentrations.

### 2.4. Data Analysis Methods

To investigate the air mass origins of the air pollutants arriving at SDZ station, 72 h backward trajectories (including 2:00, 8:00, 14:00, and 20:00 Universal Time Coordinated (UTC)) were calculated at an elevation of 500 m for every day of 2015 using the HYbrid Single-Particle Lagrangian Integrated Trajectory (HYSPLIT) 4 model (NOAA, Silver Spring, MD, USA) with meteorological input data from FNL (final operational global analysis). Potential source contribution function (PSCF) was also used to identify possible regional sources based on the HYSPLIT model (2:00 UTC). The zone of backward trajectories is divided into *i* × *j* small equal grid cells. The PSCF value in the *ij*th cell is estimated by m*_ij_*/n*_ij_*, which represents the probability of a “polluted” source from the *ij*th grid cell. Here, n*_ij_* is defined as the number of endpoints that fall in the *ij*th grid cell and m*_ij_* is defined as the number of “polluted” trajectory endpoints in the *ij*th grid cell. The resolution of the grid cell is 1° × 1°.

## 3. Results and Discussion

### 3.1. General Patterns of PM_2.5_ and Its Chemical Composition

The annual average PM_2.5_ mass concentration was 53 ± 36 μg·m^−3^ at SDZ station in 2015 (Table 1), which was 51% higher than the NAAQS for annual PM_2.5_ (35 μg·m^−3^). The meteorological data including precipitation and sunshine duration in 2015 were only slightly higher than those in 2009–2010. Although meteorological conditions in 2015 were in favor of the diffusion or scavenging of air pollutants, the annual average PM_2.5_ mass concentration in 2015 was 26% lower than the filter-based measurements (72 μg·m^−3^) during 2009–2010 [14]. Meanwhile, the annual average PM_2.5_ in BTH also evidently decreased from 98 μg·m^−3^ in 2013 to 76 μg·m^−3^ in 2015, suggesting a similar temporal trend between SDZ and BTH. Compared to other regional sites in China, the annual average PM_2.5_ at SDZ was slightly lower than at Lin’an (60 μg·m^−3^, 138 m a.s.l.) in YRD [19], slightly higher than that at a mountain site, Jinsha (49 μg·m^−3^, 750 m a.s.l.), in Hubei province [20], but was significantly higher than at a coastal regional site, HokTsui (25 μg·m^−3^, <10 m a.s.l.), in PRD [21]. The spatial patterns of PM_2.5_ between these regional sites were similar to those between the urban sites in these regions [22], suggesting the representativeness of the regional sites in the regional characteristics of PM_2.5_ pollution.

Different PM_2.5_ seasonal patterns were found between 2009 and 2010 (typical four months) filter measurements and 2005–2007 online data at SDZ [14,16]. Seasonal average PM_2.5_ concentration was higher in spring and summer than autumn and winter in previous studies, which was caused by frequent southern winds in spring and summer since the majority of air pollutant sources were located south of BTH [23]. However, a different seasonal pattern was found in this study, with higher concentrations in spring (63 ± 38 μg·m^−3^) and winter (57 ± 42 μg·m^−3^) and lower concentrations in autumn (50 ± 36 μg·m^−3^) and summer (42 ± 22 μg·m^−3^) (Table 1). The higher seasonal average PM_2.5_ observed in summer in previous studies was mainly due to the regional transport from southern urban regions and surrounding biomass burning activities [16]. Since then, significant reductions in PM_2.5_ mass concentration were observed due to decreased emission intensities in the areas south of BTH. For example, PM_2.5_ decreased by 31 μg·m^−3^ in Hebei province, 26 μg·m^−3^ in Tianjin, and 10 μg·m^−3^ in Beijing during 2013–2015. Especially notable, annual PM_2.5_ mass concentration in Shijiazhuang decreased from 149 μg·m^−3^ in 2013 to 87 μg·m^−3^ in 2015.

Water-soluble inorganic ions accounted for 34% ± 15% of PM_2.5_ mass on annual average without evident seasonal variation (31%–36%) in 2015 at SDZ. SO_4_^2−^, NO_3_^−^, and NH_4_^+^ (SNA) were the dominant water-soluble inorganic ions, accounting for 77% ± 16% of the total ions mass. The annual average SO_4_^2−^, NO_3_^−^, and NH_4_^+^ were 38%, 48%, and 16%, respectively, lower in 2015 than in 2009–2010 [14]. The reduction efficiency was 39% for SNA, higher than the 26% for PM_2.5_ in the past five years at SDZ. Meanwhile, the annual average SO_2_, NO_2_, and O_3_ in 2015 in BTH were reduced by 47%, 15%, and 2%, respectively, compared with those in 2013. Generally, the formation of SO_4_^2−^ was mainly related with heterogeneous reactions of SO_2_. NO_3_^−^ originated from heterogeneous reactions between HNO_3_ and NH_3_ during the daytime and hydrolysis ofN_2_O_5_ during the nighttime. The formation of HNO_3_ and N_2_O_5_ were related with the oxidation of NO*_x_*. The reductions of SO_4_^2−^ and NO_3_^−^ should mainly be attributed to the reductions of their respective gaseous precursors.

**Figure 2:**
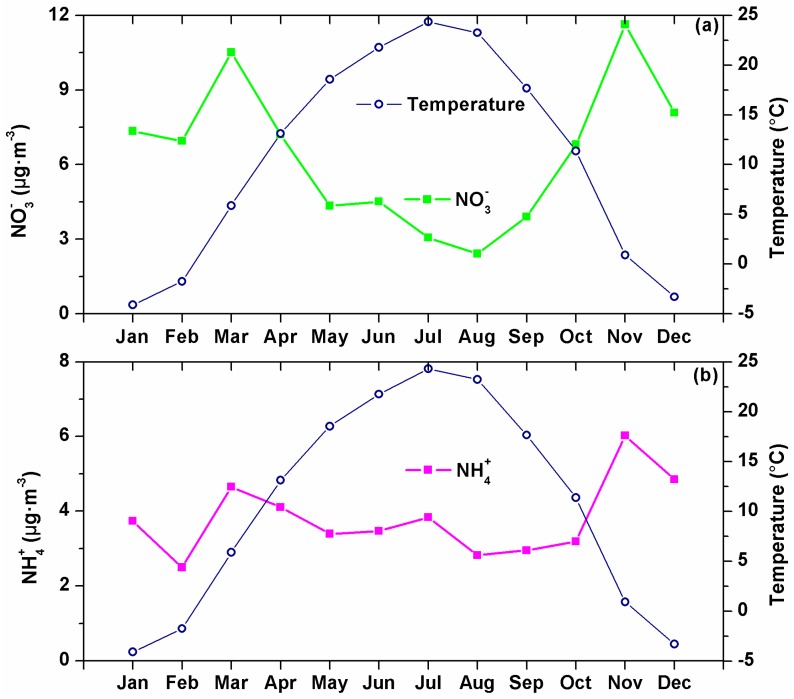
Relationships between NO_3_^−^ and ambient temperature (**a**) and NH_4_^+^ and ambient temperature (**b**).

Seasonal variations in SO_4_^2−^ and NH_4_^+^ concentrations were smaller than 30%, but more than a factor of 2.0 in NO_3_^−^ concentrations. The highest and lowest seasonal concentrations of SO_4_^2−^ were observed in summer and winter, respectively, mainly due to the different air mass origins in different seasons as discussed below in Section 3.3. In contrast, both NO_3_^−^ and NH_4_^+^ were the lowest in summer due to high ambient temperature, and differed little in the other seasons. As expected, opposite trends were seen from monthly variations between temperature and NO_3_^−^ or NH_4_^+^ (Figure 2). Higher NH_3_ emissions and high temperatures in summer, with the former increasing and the latter decreasing NH_4_^+^, resulted in the much smaller seasonal variation in NH_4_^+^ than in NO_3_^−^ [24]. Apparently, seasonal-varying emission intensities, meteorology conditions, and air mass origins all affect the seasonal variations of the ions discussed above.

**Figure 3:**
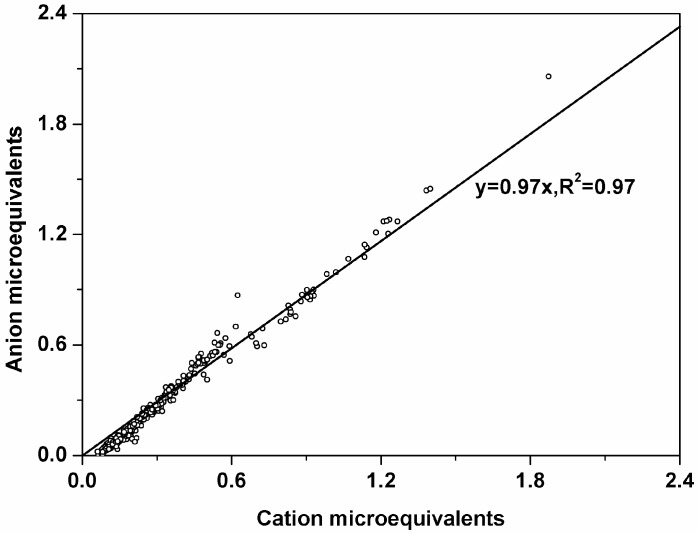
Total anions versus total cations.

**Figure 4:**
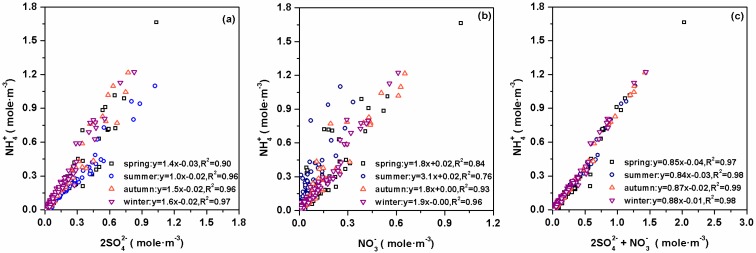
Scatter plots of NH_4_^+^ vs. SO_4_^2−^ (**a**); NH_4_^+^ vs. NO_3_^−^ (**b**); and NH_4_^+^ vs. SO_4_^2−^ plus NO_3_^−^ (**c**) in four seasons.

To calculate the ionic balance of PM_2.5_, we converted the ion mass concentrations into micro equivalents. A good correlation between anion and cation equivalents was observed (Figure 3), which indicated that the nine ionic species were the major ions in PM_2.5_. The equivalent ratios of lower loading were positioned above 1.0 and higher loading under 1.0, which suggested that PM_2.5_ in SDZ was alkaline when ions concentrations were low and was acidic when ions concentrations were higher. Good correlations were found between NH_4_^+^ and SO_4_^2−^ in all the seasons (*R*^2^ > 0.90) and between NH_4_^+^ and NO_3_^−^ in all (*R*^2^ > 0.84) but the summer season (*R*^2^ = 0.76) (Figure 4a,b). This dissociation constant of nitrate solid phase can be observed as being a function of temperature. Higher temperatures correspond to higher values of the dissociation constant. Higher temperatures shift the equilibrium of the system toward the gaseous phase. Thus, the lower correlation between NH_4_^+^ and NO_3_^−^ in summer than in other seasons was due to the higher ambient temperature in summer. In contrast, higher correlations (*R*^2^ > 0.96) were found between NH_4_^+^ and the sum of SO_4_^2−^ and NO_3_^−^ in all the seasons with regression slopes ranging from 0.84 to 0.88 (Figure 4c). These results suggested that NH_4_^+^ mainly associated with both SO_4_^2−^ and NO_3_^−^. According to the thermodynamic equilibrium model (ISORROPIA II), NH_4_^+^ was insufficient to neutralize both SO_4_^2−^ and NO_3_^−^, and other cations (e.g., Na^+^, K^+^, and Ca^2+^) were also associated with these two anions (Fountoukis and Nenes, 2007) [25].

OC and EC were important fractions of PM_2.5_, accounting for 17% ± 5% and 3% ± 1%, respectively, of PM_2.5_ mass on annual average. The converting factor between organic matter (OM) and OC was chosen to be 1.8 due to the evident effect of biomass burning in BTH [5,8]. On annual average, carbonaceous aerosols (OM + EC) accounted for 33% ± 9% of PM_2.5_ mass. The annual average concentration of carbonaceous aerosols was 27% lower than that measured during 2009–2010 [14], similar to the magnitude of PM_2.5_ reduction during the same period (26%).

From the above discussions it can be seen that the percentage reduction of PM_2.5_ was similar to that of carbonaceous aerosols, higher than that of NH_4_^+^, but much lower than those of SO_4_^2−^ and NO_3_^−^. Thus, only reducing SO_2_ and NO*_x_* emissions may not be very effective in reducing PM_2.5_ in areas with NH_3_ limited conditions, such as the case presented here. More attention should be paid to NH_3_ emission control in the future.

**Figure 5:**
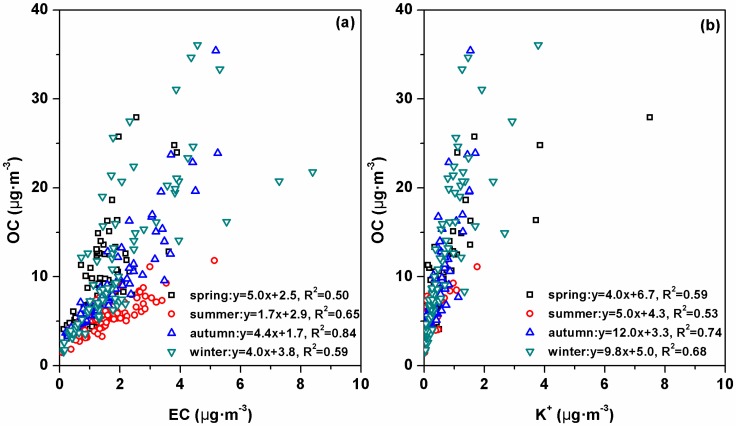
Scatter plots of OC (organic carbon) vs. EC (elemental carbon) (**a**) and OC and K^+^ (**b**) in four seasons.

Seasonal average OC concentrations were the highest in winter and the lowest in summer with seasonal variations up to a factor of 2.0. In contrast, seasonal variations of EC concentrations were smaller with seasonal average concentrations ranging from 1.3 μgC·m^−3^ in spring to 1.9 μgC·m^−3^ in winter. The correlation between OC and EC has been frequently used to identify possible sources of carbonaceous aerosols (e.g., biomass burning, coal combustion, and vehicle exhaust) [26]. Moderate correlations (with *R*^2^ ranging from 0.50 to 0.65) in spring, summer, and winter and a good correlation (*R*^2^ = 0.84) in autumn were found between OC and EC (Figure 5a). The slope in summer was 1.7, which was evidently lower than that in spring (5.0), autumn (4.4), and winter (4.0). Good correlations between OC and K^+^ were also found in spring, autumn, and winter, but not in summer (Figure 5b). The inorganic tracer K^+^ has also been used extensively to identify biomass burning. Generally, significant amounts of OC in ambient aerosols were associated with biomass burning emissions. Thus, the higher OC concentrations in summer should be somewhat related to the higher K^+^. The MODIS fire maps for BTH (Figure S1) showed much stronger open biomass burning activities in spring, summer, and autumn than in winter. Thus, biomass burning should be an important source of carbonaceous aerosols at SDZ station in most seasons including summer. Based on the regression equations between OC and EC (Figure 5a), the intercept fractions of the regression equations representing the non-combustion OC accounted for 29% of OC, which suggested that the primary OC emissions (e.g., plant detritus, resuspension of other biogenic material) were also important sources for OC at SDZ.

On annual average, the reconstructed PM_2.5_ from the determined chemical components, including water-soluble inorganic ions and carbonaceous aerosols, accounted for 67% ± 15% of measured PM_2.5_ mass. The reconstruction efficiency ranged from 63% ± 15% in spring to 70% ± 17% in winter. The lowest reconstruction efficiency in spring was likely due to the frequent fugitive dust events with high Ca^2+^ concentrations (Table 1).

### 3.2. Comparisons of Chemical Compositions under Different PM_2.5_ Pollution Levels

**Figure 6:**
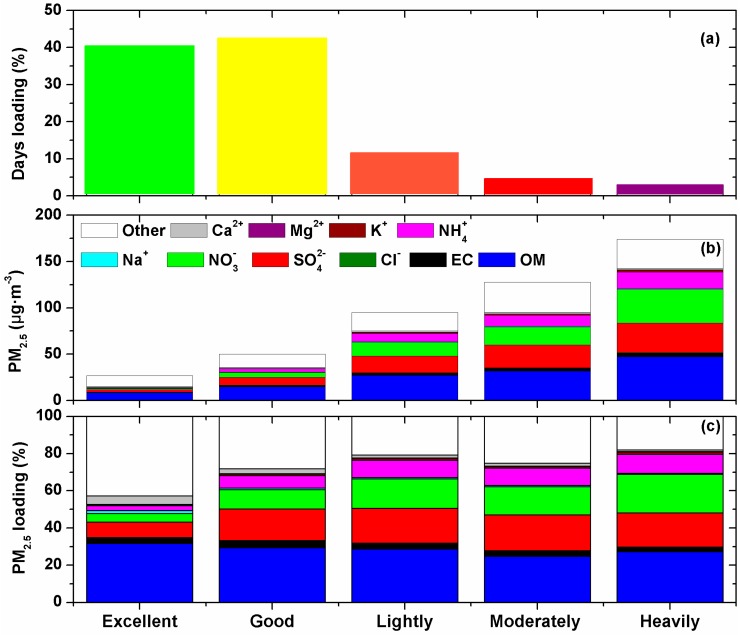
Percentage of days (**a**); mass concentrations of major chemical components in PM_2.5_ (**b**); and mass fractions of major chemical components in PM_2.5_ (**c**) under different pollution levels at SDZ station.

According to China’s Air Quality Index (AQI) (HJ633-2012), daily PM_2.5_ mass concentration threshold values can be defined as follows: <35, 35–75, 75–115, 115–150, and >150 μg·m^−3^ for excellent, good, lightly polluted, moderately polluted, and heavily polluted air quality, respectively, which accounted for 40%, 42%, 11%, 4%, and 3%, respectively, of the days in 2015 at SDZ (Figure 6a). On a seasonal scale, the total polluted days including lightly, moderately, and heavily polluted conditions accounted for 25%, 6%, 15%, and 28% in spring, summer, autumn, and winter, respectively. The higher seasonal average PM_2.5_ in spring and winter should be due to the more frequent polluted days.

The concentrations of SNA and carbonaceous aerosols in PM_2.5_ increased from 4.2 ± 2.9 μg·m^−3^ to 85.9 ± 22.4 μg·m^−3^ and from 9.2 ± 2.8 μg·m^−3^ to 51.2 ± 14.1 μg·m^−3^, respectively, when PM_2.5_ AQI increased from excellent to heavy polluted cases (Figure 6b). The mass concentrations of SO_4_^2−^ were higher than NO_3_^−^ in all the cases except during the heavily polluted days. The contributions of SO_4_^2−^, NO_3_^−^, and NH_4_^+^ to PM_2.5_ mass evidently increased from 8% ± 4%, 4% ± 3%, and 3% ± 2%, respectively, during the excellent days, to 19% ± 7%, 21% ± 4%, and 10% ± 4%, respectively, during the heavily polluted days (Figure 6c). In contrast, the contributions of carbonaceous aerosols to PM_2.5_ decreased from 34% ± 6% to 29% ± 7%, because carbonaceous aerosols increased at much slower rates than SNA when air quality changed from excellent to heavy polluted. The contributions of Cl^−^, Na^+^, K^+^, and Mg^2+^ were mostly less than 1% and changed little under different PM_2.5_ AQI. In contrast, the contribution of Ca^2+^ gradually decreased from 5% ± 1% to 1% ± 0% when PM_2.5_ AQI increased. Thus, the polluted days of PM_2.5_ were mostly caused by the rapid increase of SNA, especially NO_3_^−^ at SDZ.

On a seasonal scale, the concentrations of SNA in PM_2.5_ under polluted conditions (including lightly, moderately, and heavily polluted days) were 4.7, 5.3, 6.1, and 5.7 times of those under clean conditions (including excellent and good days) in spring, summer, autumn, and winter, respectively; while 2.0, 1.9, 2.8, and 3.1 times, respectively, for carbonaceous aerosols. Evidently, SNA increased much faster than carbonaceous aerosols when PM_2.5_ AQI increased from excellent to heavy polluted cases.

The percentage contributions of SNA to PM_2.5_ mass evidently increased from 23% ± 12%, 28% ± 17%, 24% ± 15%, and 20% ± 11% during the clean days to 42% ± 12%, 63% ± 9%, 53% ± 6%, and 39% ± 11% during the polluted days in spring, summer autumn, and winter, respectively; while for carbonaceous aerosols, decreased from 32% ± 9%, 30% ± 6%, 36% ± 6%, and 39% ± 10% to 24% ± 5%, 19% ± 3%, 30% ± 5%, and 38% ± 8%, respectively. The larger increases in the percentage contributions of SNA in summer and autumn than in spring and winter were likely caused by higher relative humidity (RH) in summer and autumn (Table 1), which favored the formation of SNA as shown in earlier studies in Beijing [27,28].

### 3.3. Potential Source Regions of PM_2.5_

**Figure 7:**
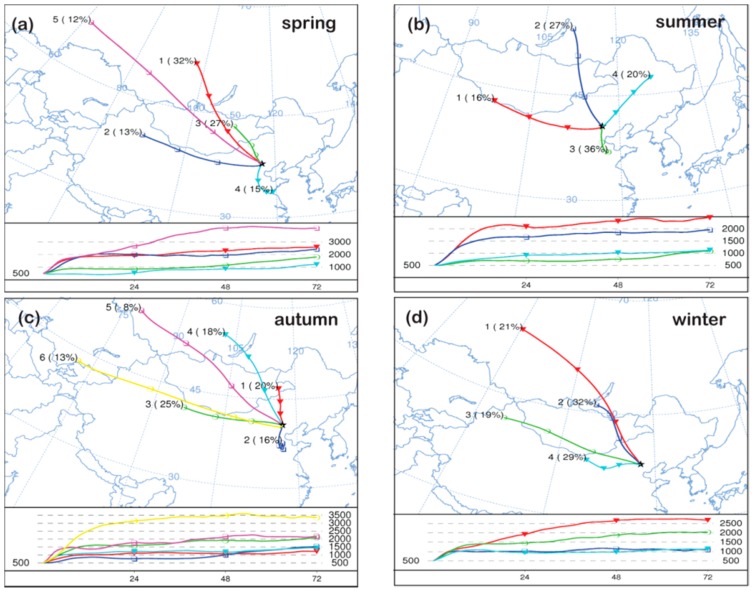
Analytical results of 72 hair mass back trajectories arriving at 500 m elevation at SDZ station in spring (**a**); summer (**b**); autumn (**c**) and winter (**d**).

The polluted days for PM_2.5_ at SDZ should be undoubtedly caused by regional transport since it is a remote rural site [16]. Trajectory clusters analysis showed similar air mass origins in spring and autumn, when air masses mainly originated from the northwest to west directions and small fractions (15%–16%) from the south (Figure 7). However, the air mass paths were slightly different between the two seasons with spring air masses passing through more polluted areas, which explained the somewhat (26%) higher concentration of PM_2.5_ in spring than in autumn. It is notable that most dominant chemical components were at similar levels in the two seasons, except SO_4_^2−^ and Ca^2+^ which were higher in spring than in autumn. This suggested that fugitive dust or dust storms were the excessive source of PM_2.5_ in spring due to low RH.

Air mass paths in summer originated from four directions including south, northeast, north, and west. The frequency of air masses from south of BTH was 36% in summer, where many air pollutants (e.g., SO_2_, NO*_x_*, OC, and EC or black carbon) sources were located [29,30,31,32]. Although the lowest concentrations of PM_2.5_ were observed in summer, the highest concentrations of SO_4_^2−^ were also observed in summer. In fact, the highest concentrations of SO_4_^2−^ were also observed in summer in several megacities (Beijing, Tianjin, and Shijiazhuang) located in the south region of BTH [14]. The concentrations of SO_4_^2−^ gradually decreased from southern cities (Tianjin and Shijiazhuang) to a northern city (Beijing) within the region. As discussed in Section 3.1, the lowest seasonal concentrations of NO_3_^−^ were observed in summer due to the highest ambient temperatures. Moreover, the lowest concentrations of carbonaceous aerosols were also observed in summer, suggesting residential heating and biomass burning instead of industrial sources south of BTH were the crucial sources of carbonaceous aerosols.

In contrast, air mass paths in winter mainly originated from northwest and west directions, and none were from the south. The lowest seasonal SO_4_^2−^ concentrations in winter thus resulted from clean air masses from the west areas of BTH (the north of Shanxi province and Inner Mongolia). The highest seasonal OC and EC concentrations in winter were probably caused by regional heating sources, as supported by the fact that the highest monthly average OC and EC were observed in the heating season from the middle of November to the middle of March next year (Table S1). In fact, the highest concentrations of carbonaceous aerosols were also observed in winter at urban sites in BTH [33]. The spatial distributions of carbonaceous aerosols were different from those of SO_4_^2−^ presented in a previous study [14]. The concentrations of carbonaceous aerosols in Chengde, a small city in BTH, were higher than those in megacities (e.g., Beijing and Tianjin), suggesting that local residential heating surrounding the SDZ station might have also contributed to the observed carbonaceous aerosols.

The above discussions suggest that different air mass origins/paths in different seasons resulted in the different seasonal variations of major chemical components in PM_2.5_ at SDZ. In particular, higher concentrations of SO_4_^2−^ were mainly associated with air masses that originated from south of BTH where major SO_2_ emission sources were located, while higher concentrations of carbonaceous aerosols were mainly caused by the regional-scale heating activities.

**Figure 8:**
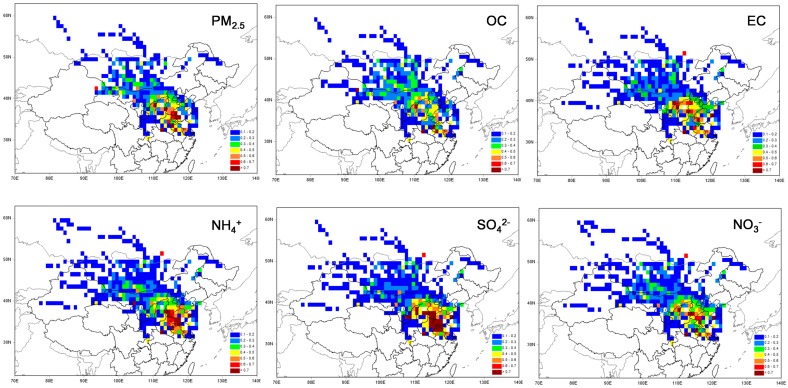
The potential source contribution function (PSCF) values for PM_2.5_, SNA (sulfate, nitrate, and ammonium), and carbonaceous aerosols at SDZ station.

The PSCF model was run to further investigate the source regions of PM_2.5_ and its major components (including OC, EC, SO_4_^2−^, NO_3_^−^, and NH_4_^+^). Although the locations of the higher values or “polluted spots” were different for PM_2.5_ and the dominant chemical components, the “polluted spots” mainly originated from the south to west directions (Figure 8). The spatial distributions of the “polluted spots” mainly covered the areas bordering Shandong, Henan, and Hebei provinces, where major emission sources of NO*_x_*, SO_2_, and carbonaceous aerosols were situated in China [29,30,31,32]. As expected, the spatial distributions of “polluted spots” for PM_2.5_ and SNA were similar because the contributions of SNA to PM_2.5_ were higher than that of carbonaceous aerosols, especially on polluted days. Although the annual mass concentration of NO_3_^−^ was much higher than NH_4_^+^ and slightly lower than SO_4_^2−^, the “polluted spots” for NO_3_^−^ were evidently less than those of SO_4_^2−^ and NH_4_^+^. Clearly, the “polluted spots” of SO_4_^2−^ and NH_4_^+^ were mainly associated with air masses from the south directions of SDZ. Air masses from the south were more frequent in summer than in other seasons. However, higher ambient temperatures in summer were conducive to the evaporation of NO_3_^−^, causing fewer “polluted spots” for NO_3_^−^ than SO_4_^2−^ and NH_4_^+^.

The spatial distributions of “polluted spots” were slightly different between OC and EC, although most of the “polluted spots” were located in Hebei, Shanxi, and Shandong provinces where high emissions of black carbon existed in China (Qin and Xie, 2012) [31]. Other “polluted spots” of EC were also found in the areas bordering Shanxi and Inner Mongolia located west of SDZ (Qin and Xie, 2012) [31]. In addition, moderate values of OC were also observed within Mongolia and Inner Mongolia. Evidently, a portion of OC and EC came from the different regions, which decreased the correlation between OC and EC. In most cases, PM_2.5_ and the dominant chemical components at SDZ were mainly associated with the regional transport from Hebei, Henan, Shandong, and Shanxi provinces.

## 4. Conclusions

PM_2.5_ pollution seemed to have alleviated in BTH in recent years, as seen from the 26% decrease in the annual average PM_2.5_ and 34% decrease in the sum of SNA and carbonaceous aerosols at SDZ station in 2015 compared to those during 2009–2010. However, the annual PM_2.5_ mass concentration at SDZ was still 51% higher than the NAAQS in 2015, which was largely caused by high concentrations of NO_3_^−^, SO_4_^2−^, and NH_4_^+^ during polluted days, especially the increase of NO_3_^−^ during heavily polluted days. Regional transport of air pollutants originating from the south to west regions of SDZ was the most likely source of PM_2.5_, especially SO_4_^2−^. Open biomass burning activities and heating (residential coal combustion) in BTH likely contributed to the high concentrations of carbonaceous aerosols at SDZ station. While there is a need to further decrease NO*_x_* and SO_2_ emissions in order to reduce the number of heavily polluted days in this region, decreasing NH_3_ emissions might be more effective since the area is under a NH_3_-limited scenario in the formation of PM_2.5_. Additional modeling sensitivity studies are needed to investigate the non-linearity between gaseous-precursors emissions reduction and PM_2.5_ formation in this region for providing scientific guidance on making future emission control policies.

## Figures and Tables

**Table 1:** Statistics of the determined chemical compositions in PM_2.5_ and selected meteorological factors in Shangdianzi (SDZ).

	PM_2.5_	OC	EC	Na^+^	NH_4_^+^	K^+^	Mg^2+^	Ca^2+^	Cl^−^	SO_4_^2−^	NO_3_^−^	Temp	RH	PR	SD
	(μg·m^−3^)											(°C)	(%)	(mm)	(h)
Annual, 2015	53 ± 36	8.6 ± 6.0	1.6 ± 1.1	0.4 ± 0.3	3.8 ± 4.7	0.5 ± 0.5	0.1 ± 0.1	1.4 ± 0.4	0.2 ± 0.4	8.5 ± 9.2	6.4 ± 8.3	11 ± 11	54 ± 21	522	2586
Spring, 2015	63 ± 38	9.0 ± 4.8	1.3 ± 0.7	0.5 ± 0.2	4.1 ± 5.1	0.5 ± 0.6	0.1 ± 0.2	1.7 ± 0.5	0.2 ± 0.2	9.1 ± 9.5	7.5 ± 9.3	13 ± 7	39 ± 17	85	789
Summer, 2015	42 ± 22	5.5 ± 1.9	1.5 ± 0.9	0.5 ± 0.3	3.4 ± 4.0	0.2 ± 0.3	0.0 ± 0.0	1.2 ± 0.1	0.1 ± 0.1	9.6 ± 10.1	3.3 ± 3.9	23 ± 3	66 ± 14	283	738
Autumn, 2015	50 ± 36	8.6 ± 5.7	1.6 ± 1.2	0.4 ± 0.3	4.0 ± 5.2	0.4 ± 0.4	0.0 ± 0.0	1.3 ± 0.1	0.2 ± 0.1	7.8 ± 9.0	7.3 ± 9.4	10 ± 8	67 ± 17	148	530
Winter, 2015	57 ± 42	11.3 ± 8.2	1.9 ± 1.6	0.4 ± 0.3	3.7 ± 4.6	0.6 ± 0.7	0.1 ± 0.2	1.3 ± 0.3	0.4 ± 0.8	7.4 ± 8.0	7.5 ± 8.9	−3 ± 3	45 ± 19	7	529
Annual, 2009–2010	72 ± 48	10.8 ± 6.8	3.9 ± 1.9	0.3 ± 0.1	4.5 ± 3.7	1.2 ± 1.1	0.1 ± 0.1	0.7 ± 0.7	0.8 ± 1.1	13.8 ± 14.9	12.2 ± 14.3	11	53	399	2494

OC: Organic carbon, EC: Elemental carbon, Temperature: Temp; Relative humidity: RH; Precipitation (PR): sum of precipitation; Sunshine duration (SD): sum of sunshine duration.

## Supplementary Materials

The following are available online at www.mdpi.com/1660-4601/13/12/1202/s1, Figure S1: Moderate-resolution Imaging Spectroradiometer fire maps for Beijing-Tianjin-Hebei in four seasons (red dots represent field open fire dots), Table S1: Monthly averages of the determined chemical compositions in PM_2.5_ and selected meteorological factors in Shangdianzi (SDZ).
