# Supplementary material for: High Contributions of Secondary Inorganic Aerosols to PM2.5 under Polluted Levels at a Regional Station in Northern China
Source: Int J Environ Res Public Health. 2016 Dec 15;13(12):1202. doi: 10.3390/ijerph13121202 (PMC5201343; doi:10.3390/ijerph13121202)
Supplement: Supplementary file 1 [file ijerph-13-01202-s001.pdf]

Supplementary Materials: High Contributions of Secondary Inorganic Aerosols to $PM_{2.5}$ under Polluted Levels at a Regional Station in Northern China

Yang Li, Jun Tao, Leiming Zhang, Xiaofang Jia and Yunfei Wu

Table S1. Monthly averages of the determined chemical compositions in $PM_{2.5}$ and selected meteorological factors in Shangdianzi (SDZ).

	$PM_{2.5}$	OC	EC	Na^+	NH_4^+	K^+	Mg^{2+}	Ca^{2+}	Cl^-	SO_4^{2-}	NO_3^-	Temp.	RH	PR	SD
						(μg·m^{-3})						(°C)	(%)	(mm)	(h)
January	55 ± 39	11.8 ± 8.1	1.9 ± 1.5	0.4 ± 0.3	3.7 ± 4.7	0.7 ± 0.6	0.0 ± 0.0	1.3 ± 0.2	0.4 ± 0.2	6.5 ± 7.3	7.3 ± 8.4	−4 ± 2	38 ± 15	0	191
February	57 ± 48	10.1 ± 9.2	1.6 ± 1.7	0.5 ± 0.3	2.5 ± 3.1	0.8 ± 1.0	0.2 ± 0.3	1.4 ± 0.5	0.5 ± 1.3	7.1 ± 7.2	6.9 ± 8.8	−2 ± 3	38 ± 17	4	175
March	67 ± 50	11.4 ± 6.3	1.3 ± 0.7	0.7 ± 0.2	4.7 ± 6.5	0.8 ± 0.9	0.1 ± 0.2	1.5 ± 0.3	0.4 ± 0.2	8.8 ± 10.6	10.5 ± 13.5	6 ± 6	30 ± 14	1	258
April	62 ± 37	7.9 ± 3.1	1.4 ± 0.7	0.4 ± 0.2	4.1 ± 4.4	0.4 ± 0.3	0.1 ± 0.2	1.7 ± 0.6	0.1 ± 0.1	8.9 ± 7.2	7.2 ± 6.6	13 ± 5	42 ± 18	49	252
May	58 ± 22	7.3 ± 3.0	1.2 ± 0.6	0.4 ± 0.2	3.4 ± 3.9	0.3 ± 0.2	0.2 ± 0.1	1.8 ± 0.6	0.1 ± 0.1	9.6 ± 9.5	4.3 ± 3.1	19 ± 4	44 ± 16	35	279
June	43 ± 30	5.2 ± 2.4	1.3 ± 0.9	0.7 ± 0.2	3.5 ± 5.2	0.3 ± 0.4	0.1 ± 0.0	1.3 ± 0.1	0.1 ± 0.1	9.4 ± 12.3	4.5 ± 5.6	22 ± 2	61 ± 14	58	220
July	47 ± 15	6.1 ± 1.1	2.1 ± 0.6	0.3 ± 0.2	3.8 ± 3.0	0.2 ± 0.1	0.0 ± 0.0	1.2 ± 0.1	0.1 ± 0.0	11.6 ± 8.9	3.1 ± 2.3	24 ± 2	66 ± 15	146	259
August	37 ± 19	5.1 ± 1.8	1.2 ± 0.9	0.6 ± 0.2	2.8 ± 3.6	0.2 ± 0.1	0.1 ± 0.0	1.2 ± 0.1	0.1 ± 0.1	7.7 ± 8.8	2.4 ± 3.0	23 ± 2	71 ± 10	79	259
September	40 ± 21	6.2 ± 2.0	1.2 ± 0.9	0.6 ± 0.2	2.9 ± 3.8	0.3 ± 0.2	0.0 ± 0.0	1.2 ± 0.1	0.1 ± 0.1	7.3 ± 8.5	3.9 ± 4.2	18 ± 2	73 ± 11	92	203
October	47 ± 39	8.0 ± 5.3	1.4 ± 1.3	0.3 ± 0.2	3.2 ± 5.1	0.5 ± 0.5	0.0 ± 0.0	1.3 ± 0.2	0.2 ± 0.1	5.9 ± 8.6	6.8 ± 9.6	11 ± 4	58 ± 18	10	236
November	66 ± 42	11.9 ± 7.2	2.1 ± 1.3	0.3 ± 0.3	6.0 ± 6.1	0.6 ± 0.4	0.0 ± 0.0	1.2 ± 0.1	0.2 ± 0.2	10.5 ± 9.6	11.6 ± 11.5	1 ± 6	71 ± 16	45	91
December	59 ± 40	12.0 ± 7.5	2.2 ± 1.4	0.3 ± 0.3	4.9 ± 5.5	0.5 ± 0.4	0.0 ± 0.0	1.2 ± 0.1	0.3 ± 0.2	8.5 ± 9.4	8.1 ± 9.7	−3 ± 2	61 ± 17	3	163

Temperature: Temp.; Relative humidity: RH; Precipitation (PR): sum of precipitation; Sunshine duration (SD): sum of sunshine duration.

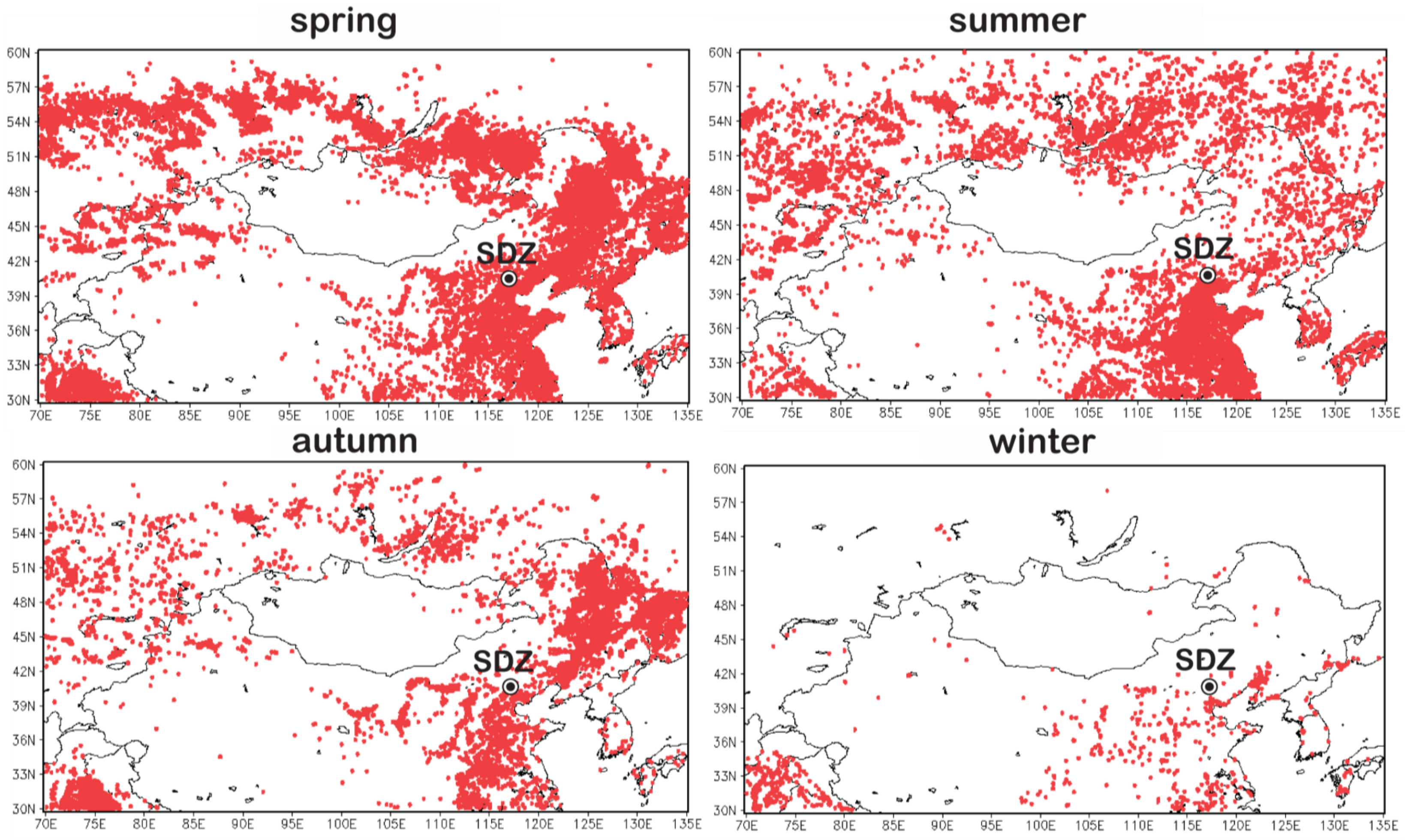

Figure S1. Moderate-resolution Imaging Spectroradiometer fire maps for Beijing-Tianjin-Hebei in four seasons (red dots represent field open fire dots).

© 2016 by the authors. Submitted for possible open access publication under the terms and conditions of the Creative Commons Attribution (CC-BY) license (http://creativecommons.org/licenses/by/4.0/).
